# DEVELOPING A QUALITY-OF-RELATIONSHIP INTERVENTION FOR STROKE-SURVIVOR FAMILY CAREGIVER DYADS

**DOI:** 10.1093/geroni/igz038.1036

**Published:** 2019-11-08

**Authors:** Michael J McCarthy, Karen Lyons, Dorothy Dunn, Yolanda Garcia, Tamilyn Bakas

**Affiliations:** 1 Northern Arizona University, Flagsttaff, Arizona, United States; 2 Boston College William F. Connell School of Nursing, Chestnut Hill, Massachusetts, United States; 3 University of Cincinnati, Cincinnati, Ohio, United States

## Abstract

A strong interpersonal relationship after stroke is important for the well-being of survivors and family caregivers. At present, few interventions are specifically designed to strengthen the relationship between members of the care dyad. The aim of this study is to develop, validate, and pilot test a quality of relationship intervention for stroke dyads. This poster presents findings from the content validity phase of the study and includes “tips” that dyads offered for maintaining a strong relationship. Semi-structured interviews were conducted with N=19 dyads to solicit information about relationship problems and tips. These data were used to develop a 17-item relationship assessment questionnaire with 17 “tip sheets” corresponding to each item. A Delphi process was used to obtain feedback from a 10-member expert panel about the degree to which the questionnaire and each tip sheet was (1) relevant; (2) clear; (3) accurate, and; (4) useful. Expert agreement ranged for 80% to 100%. The final materials included tips for dealing with issues that arise when one or both partners experience communication, mobility, cognitive, or emotional issues; reaching agreement about different aspects of recovery; working together to meet each partners’ needs; adjusting to new roles after stroke; dealing with relationship problems that predated stroke, and; getting support from other family members, friends, peers, and professionals. Findings highlight areas to consider in promoting strong relationships in care dyads. Future research is needed to examine whether these materials can help to reduce stressful interactions for care dyads, promote dyadic coping, and improve overall relationship quality.

